# Development of a Scale Measuring Emotional Catharsis through Illness Narratives

**DOI:** 10.3390/ijerph18168267

**Published:** 2021-08-04

**Authors:** Hung-Chang Liao, Ya-huei Wang

**Affiliations:** 1 Department of Health Policy and Management, Chung Shan Medical University, Taichung 40201, Taiwan; hcliao@csmu.edu.tw; 2 Department of Medical Management, Chung Shan Medical University Hospital, Taichung 40201, Taiwan; 3 Department of Applied Foreign Languages, Chung Shan Medical University, Taichung 40201, Taiwan; 4 Department of Medical Education, Chung Shan Medical University Hospital, Taichung 40201, Taiwan

**Keywords:** catharsis, medical care professionals and students, purification

## Abstract

Objective: This study intended to construct a scale measuring the catharsis effect on medical professionals or students through illness narratives (ECS-IN). Methods: After a systematic literature review and panel discussion, the researchers conducted a pilot study with a sample of seven hundred and eighty-two randomly selected healthcare students and providers in Taiwan to examine psychometric properties using exploratory factor analysis (EFA) and confirmatory factor analysis (CFA) for item derivation and factor extraction. The researchers also tested the validities and reliabilities of the ECS-IN scale to confirm its feasibility. Results: the EFA yielded 29 items and three factors: “emotional identification as self-healing” (12 items; 55.500% of variance explained), “emotional release for compensation” (10 items; 7.465% of variance explained), and “emotional adjustment for intellectual growth” (7 items; 4.839% of variance explained). The CFA yielded an 18-item, three-factor model with satisfactory fit to the data, where the *χ*^2^/*df* ratio = 1.090, Tucker–Lewis index (TLI) = 0.996, comparative fit index (CFI) = 0.997, and root mean square of approximation (RMSEA) = 0.020. The convergent validity and discriminant validities also demonstrated the feasibility of the ECS-IN scale. For the first version of the ECS-IN scale (29 items), the Cronbach’s alphas for the three factors and the overall scale were in the range between 0.912 and 0.971; for the reduced version of the scale (18 items), the Cronbach’s alphas and composite reliabilities were in the range of 0.888–0.946 and 0.890–0.968. Conclusion: The findings proved that the ECS-IN could be a reliable and valid instrument to assess participants’ emotional catharsis through illness narratives.

## 1. Introduction

The word “catharsis,” originating from the Greek words *katharein* and *kathares,* refers to a Greek chorus’s use of music, song, and dance to achieve the cleanliness, purgation, or purification of humans’ souls or emotions [1]. The Greek philosopher Aristotle [2] used the term “catharsis” in *Poetics* with respect to dramatic arts, especially poetry and tragedies, referring to the release of pity and fear through art so as to reach purification and renewal of the soul [2]. Aristotle proposed that, when intense emotions, such as pity (*eleos*) and fear (*phobos*), are aroused, dramatic art can achieve the goal of catharsis, that is, it can bring out the purgation and purification of those intense emotions. As he [2] suggested, an audience may partake in various experiences while watching dramatic arts, perhaps evoking their pity and fear concerning a hero’s dilemma. However, while experiencing this conflict along with the tragic hero and being aroused to pity and fear, the audience may also purify their own emotions by identifying with the hero and hence releasing their inner stress and reaching purification and peace of mind [2]. Thus, it can be concluded that the word “catharsis” involves the liberation of those negative or intense feelings or effects, such as pity, anger, frustration, and so forth, so as to restore order or to bring the audience to a proper state of mental health.

The ancient Greeks also used the term “catharsis” to mean the cleansing of the body via medicine and the cleaning of the soul via art. While being used in the temples of Aesculapius, the word “catharsis” involved nocturnal acts of purification as a means to heal those who were ill [1]. In medicine, the word “catharsis” not only refers to the expectoration or discharge of any harmful material from the body, such as menstruation, diarrhea, and vomitus, but can be a discharge of any harmful or negative emotions from one’s mind or unconsciousness so as to restore one to the previous normal state or mentally balanced condition. Hippocrates made an association between catharsis and healing, identifying catharsis as an agent for purification and hence determining that it impacts illness, be it physical or mental [3].

Based on Aristotle’s tragic catharsis [2], research has also shown that tragic narratives may help readers purge their unpleasant emotions and hence further contextualize these emotions [4]. However, while people are in greater arousal of tragic emotions, they may not completely purge their negative emotions but rather develop greater tension and anxiety [5]. Some research in media violence shows that when people are exposed to violent scenarios, they may become more agitated [6,7,8]. They may increase their anger intensity via the reinforcement of negative emotions [9]. However, Scheele and DuBois’s research [10] revealed that while watching media violence, viewers may find a healthy venue to purge their aggressive and violent feelings. Though there is an inconsistency in the findings of catharsis effect, one consensus about the catharsis effect lies in its healing and restorative value [5].

The catharsis effect may also be reached through illness narratives to release negative emotions, such as fear, anxiety, or even anger. Through using illness narratives as catharsis, people can vent their emotions and hence reach a healing effect [11,12]. When people are imaginatively confronted with frustrating or grievous situations through participation in fictitious scenarios, their own internal aggression, anger, or other repressed feelings can be released, leading to a catharsis effect and the removal of those negative feelings or emotions [13]. Moreover, through the medical approach of adopting the illness narratives of patients, patients’ families, or healthcare professionals in medical education and clinical practice, healthcare providers or students can enhance their self-reflection and empathy toward those who are suffering, thereby further facilitating the healing process [14]. Indeed, recognition, interpretation, and insight into any ambivalence regarding illness stories may also lead to a catharsis effect for healthcare providers and students, causing a release of pity, fear, or stress connected to patients’ suffering or workplace stress [15]. Hence, as with Greek tragedies, illness narratives, also a kind of literary narrative, are used in medical education as a means to free oneself from excessive suffering or stress from illness in order to acquire release and comfort of mind. As Fendt [16] noted, the fictitious presentation of actions and moral choices regarding important matters not only arouses passions but also brings about a catharsis related to these passions, which, consequently, allows people to maintain equilibrium and avoid the extremes of pity, fear, anxiety, anger, or indignation.

Illness is a traumatic experience—many would say a tragic experience—as those afflicted with serious illness must contend with not only the physical painfulness but also the uncertainties of the treatment even with the use of advanced medical technology. However, those suffering from illness can write illness narratives and, through the narrating process, achieve introspection and reflection through their illness scenarios while simultaneously distancing themselves from the situation via the fictitious scenario [17]. Hence, illness narratives may bring a healing effect to those suffering from the agony of illness. In other words, illness narratives may become an emotional cataclysm to help those who are ill reach a catharsis after which they might resume a meaningful life [18].

Given the healing power of narrative writing, healthcare professionals may also find relief, albeit temporary, from the emotional stress that may come from the uncertainty of a diagnosis and prognosis through illness narratives [11,19]. Because catharsis through illness narratives allows people to express their negative responses on a deeper level, they may ultimately develop new coping skills as well as a deeper understanding of themselves and the meaning of their existence [20]. Illness narratives can also help people to cope with ethical dilemmas in clinical situations [21,22,23]. Moreover, this strategy can offer relief to those experiencing burnout or feeling overwhelmed by their daily medical or health care realities, especially during the COVID-19 pandemic. In essence, illness narratives can serve a restorative function by achieving the catharsis effect of illness storytelling when fear, horror, anger, and anxiety are brought to light [24]; by recognizing their feelings, soothing their negative emotions, and eliminating these disturbing and harmful elements, tranquility of the mind may be achieved. Moreover, after releasing their tension and negative emotions, they can further identify, manage, and solve their problems [25].

Because illness narratives can help healthcare workers achieve freedom from their work burdens or burnout, medical educators have implemented illness narratives and narrative medicine in medical education and on-the-job training to help students and professionals mitigate the possible destructive consequences, especially during the current coronavirus crisis, that have already inflicted healthcare professionals with work burden and burnout [26], especially since they have no choice but to face the tragic loss of their patients. Through the use of illness narratives involving the struggles of loss or death, healthcare providers may reach a state of catharsis and find an outlet for their dismay.

Given the power and advantages of illness narratives, it seems necessary for medical and health care students to acquire these narrative skills for illness narratives so as to reach a catharsis and thereby relieve themselves of their own pity, fear, and other emotional stress in clinical settings. However, there is no research regarding the use of illness narratives to reach an emotional catharsis for healthcare students or professionals, nor is there an emotional catharsis scale in a Chinese edition. Although there is one general emotional catharsis scale in a Turkish edition, developed by Çerkez [27] and used to examine the relationship between perfectionism and the density of experiencing catharsis, it is based on a Turkish cultural context and general college students and not on a Taiwanese cultural context and not on medical students and professionals. Moreover, being part of a doctoral dissertation, that scale is unable to be accessed, resulting in the importance of developing a scale based on the Taiwanese cultural context to measure medical care students’ and professionals’ emotional catharsis through illness narratives (ECS-IN).

## 2. Material and Methods

### 2.1. Procedure and Participants

This study intended to construct a scale measuring the emotional catharsis effect on medical professionals or students through illness narratives (ECS-IN) based on Taiwanese healthcare contexts. As there is only one emotional catharsis scale in a Turkish edition and no catharsis scale regarding illness narratives, because of a lack of catharsis measures [28] and in order to explore emotional catharsis based on illness narratives, the researchers conducted a thorough literature review on emotional catharsis, illness narratives, and narrative medicine to systematically collect scale items mainly from the following databases: ScienceDirect, Proquest, Pubmed, and EBSCO. After extensive literature review for item generation based on Tyupa’s [29] theoretical framework for back-translation process: forward translation → back-translation → back-translation review and discussion → finalization, the researchers drafted the ECS-IN scale items in a Chinese edition; then, two bilingual English teachers reviewed the drafted Chinese scale items, translated them into English scale items, and later translated them back into Chinese scale items to ensure its semantic equivalence. After going through the translation and back-translation process to ensure the quality of translation [30], to verify the face validity and content validity, the researchers convened expert panels specializing in medical education, statistics, social science, and instrument assessment for panel discussions to examine whether the scale items targeted the theoretical construct [31]. The panel sorted the collected items into three categories: “emotional identification as self-healing,” “emotional release for compensation,” and “emotional adjustment for intellectual growth.” For those items with less than 75% of agreement in their relevance to the theoretical construct [32], the panel decided to drop the items; hence, after a series of panel discussions, the 89 items were reduced to 52 items. The experts further examined whether there was any difference between the original and the subsequent Chinese scale items; after minor modifications, they reached a consensus. The ECS-IN was rated on a nine-point Likert scale, with 9 meaning “strongly agree” and 1 meaning “strongly disagree.” The higher the score, the stronger the emotional catharsis a participant had.

After the literature review and panel discussions, the researchers conducted a pilot study with a sample of seven hundred and eighty-two randomly selected healthcare students and providers in Taiwan. These students were students in a medical university with the school’s own hospital next to the campus. To facilitate their interprofessional competencies and to provide healthcare or social service based on community needs, they were required to receive service learning in healthcare settings once enrolling in the university as a freshman. These healthcare settings included medical institutions, non-profit social welfare institutions or communities, and public health bureaus or health centers. The surveys with missing data were taken to be invalid surveys, leading to a 95.269% response rate (*N* = 745) from the available participants. To examine psychometric properties, the researchers used exploratory factor analysis (EFA) and confirmatory factor analysis (CFA) for item derivation and factor extraction. The researchers also tested the validities and reliabilities of the ECS-IN scale to confirm its feasibility.

### 2.2. Statistical Analysis

The study adopted EFA and CFA statistic technologies to analyze the consistency of the ECS-IN scale factors. The study performed EFA on the sample of healthcare students (*N* = 507) to derive the first version (29 items) and performed CFA on the sample of healthcare professionals (*N* = 238) to derive the reduced version (18 items). All data were first analyzed using SPSS [33] to conduct EFA to uncover the underlying factor structure of the developed ECS-IN scale, using eigenvalues greater than 1.0, principal component analysis (PCA), and promax rotation. The study also used the Kaiser–Meyer–Olkin (KMO) test [34,35] and Bartlett’s test of sphericity [36,37] to examine the adequacy of the sample size for factor analysis [38]. In addition, factor loadings, means, standard deviations, medians, interquartile ranges, and Cronbach’s alphas were also analyzed. The researchers later performed CFA, using AMOS 24 [39] to validate the factor structure of the first version (29 items) of the scale. Additionally, the researchers used a number of fit indices to examine the goodness of fit of the reduced version (18 items) of the scale for the ECS-IN scale, including *χ*^2^/*df* ratio [40], Tucker–Lewis Index (TLI) [41], comparative fit index (CFI) [41], and root mean square error of approximation (RMSEA) [40]. In addition, the researchers tested the convergent validity of the ECS-IN scale using average variance extracted (AVE) values, which should be greater than or at least equal to 0.50. In addition, the AVE values should be smaller than the composite reliabilities [42,43]. To test the discriminant validity, the researchers compared the square root values of the average variance extracted (√AVE) with the values of the correlation coefficients (*r*) between factors [37]. The researchers also used Cronbach’s alphas and Pearson’s correlation coefficients to confirm the internal reliability within each factor and between any two factors of the scale.

### 2.3. Ethical Consideration

The study was approved by the Institutional Review Board of Chung Shang Medical University Hospital (No. CS16157) and also abiding by the guidelines of the Research Ethics Framework of Society Institute in Taiwan [44]. Before collecting the data, the researchers explained the research purpose to the participants and ensured both their anonymity and the confidentiality of their personal information.

## 3. Results

Of the 745 valid participants, 460 participants (61.74%) were female, and 285 participants were male (38.26%); 462 were aged between 19 and 22 years old (62.01%), 150 were aged between 22 and 30 years old (20.14%), and 133 were aged 30 years or over (17.85%). Of these participants, 238 participants were healthcare professionals (31.95%), and 507 participants were medical and healthcare students (68.05%). There were 105 students studying in the college of medicine (14.09%), 95 students in the college of oral medicine (12.75%), 120 students in the college of medical science and technology (16.11%), and 187 students in the college of health care and management (25.10%).

### 3.1. Exploratory Factor Analysis (EFA)

#### 3.1.1. KMO Test and Bartlett’s Test of Sphericity

**Figure 1:**
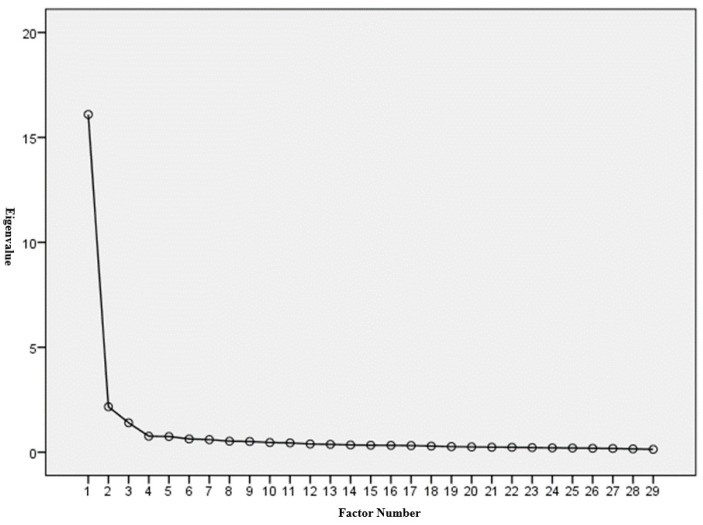
Scree plot for exploratory factor analysis of ECS-IN.

The study used the KMO test [34,35] and Bartlett’s test of sphericity [36,37] to examine the ECS-IN scale’s structure validity. The KMO value was 0.969, higher than the minimum acceptable value of 0.6 [45,46]. Bartlett’s test of sphericity was also significant (approx. chi-square = 12,712.741; degree of freedom = 406; *p* = 0.000 < 0.05). The results of the KMO test and Bartlett’s test of sphericity revealed the suitability of the sample size for meaningful EFA. The scree-plot graphic also showed that the optimum number of factors for the ECS-IN scale is three (see Figure 1).

#### 3.1.2. First Version (29 Items) for the ECS-IN Scale, Using EFA

The researchers first used EFA to elicit hidden interrelationships among items and group items that are part of unified variables or factors [47] and to verify the internal consistency and construct validity of the scale using eigenvalues greater than 1.0 and principal component analysis. Additionally, promax rotation, an oblique rotation method, was used to maximize the correlations between factors and items [48]. The items were retained if their factor loading was greater than 0.60 on the relevant factor and less than 0.60 on the nonrelevant factor.

The initial scale with 52 items was subjected to an EFA to examine the structure of the developed ECS-IN. After the EFA and principle component analysis, 29 items and three factors were identified: “emotional identification as self-healing,” “emotional release for compensation,” and “emotional adjustment for intellectual growth.” The three factors retained in the ECS-IN explained 67.803% of the variance. Factor 1 contained 12 items related to “emotional identification as self-healing” and explained 55.500% of the variance explained. Factor 2 contained 10 items related to “emotional release for compensation,” explaining 7.465% of the variance. Factor 3 contained seven items on “emotional adjustment for intellectual growth,” explaining 4.839% of the variance. All of the factor loadings were higher than 0.6, ranging between 0.62 and 0.77. Table 1 represents the eigenvalues of the three factors from the principle component analysis; all the eigenvalues of these three factors were larger than one: 16.095, 2.165, and 1.403 in “emotional identification as self-healing,” “emotional release for compensation,” and “emotional adjustment for intellectual growth,” respectively (see Table 1). These results demonstrated the multidimensionality of the ECS-IN.

#### 3.1.3. Reliability of the First Version (29 Items) for the ECS-IN Scale

The researchers also used Cronbach’s alpha to measure the internal consistency within each factor of the ECS-IN scale. The minimally acceptable Cronbach’s alpha value is 0.7 [49,50,51]. Cronbach’s alpha values between 0.93 and 0.94 are regarded as excellent; values between 0.91 and 0.93 are regarded as strong values. The collected data showed that the Cronbach’s alpha values for the three factors were 0.960, 0.938, and 0.912 (“emotional identification as self-healing,” “emotional release for compensation,” and “emotional adjustment for intellectual growth,” and the Cronbach’s alpha for the entire questionnaire was 0.971 (see Table 1). The results prove that the ECS-IN could be a reliable and valid instrument to assess participants’ emotional catharsis through illness narratives. 

#### 3.1.4. Descriptive Statistics of the First Version (29 Items) for the ECS-IN Scale

The mean score for factor 1 was 68.630 (median: 68.647; interquartile range: 60.000–80.000), the mean score for factor 2 was 55.409 (median: 55.000; interquartile range: 48.000–66.000), and the mean score for factor 3 was 44.984 (median: 46.000; interquartile range: 38.00–51.00). Table 2. shows the average item scores (*Mean*) and standard deviations (*S.D.*) of the first version (29 items) for the ECS-IN scale.

### 3.2. Confirmatory Factor Analysis (CFA)

The EFA performed on the sample of healthcare students demonstrated a three-factor structure for the ECS-IN scale. To investigate whether the established factorial structure and factor-loading pattern fitted in the medical practice context, the study further performed CFA on the sample of 238 healthcare professionals. The sample size *(N = 238)* used in the study meets Hair et al.’s minimum criterion of sample size 260 (5 times *29* items) for multivariate data analysis research [42].

#### 3.2.1. Goodness of Fit of the Reduced Version (18 Items) for the ECS-IN Scale

**Figure 2:**
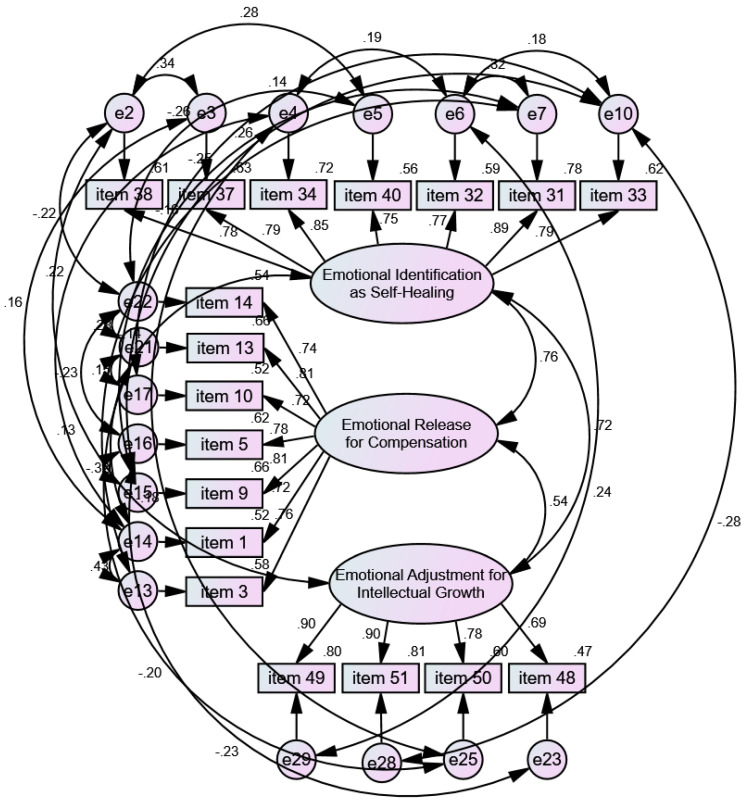
Reduced version (18 items) of the ECS-IN scale.

To examine the goodness of fit of the three-factor structure derived from the first version (29 items) of the ECS-IN scale, the researchers further used CFA, a structural equation model, to evaluate the underlying latent factor structure of the ECS-IN scale from the same sample on which the EFA was performed. The items with factor loading greater than 0.60 were retained. The reduced version yielded 18 items related to the three factors of “emotional identification as self-healing,” (7 items; factor loadings: 0.747–0.886), “emotional release for compensation,” (7 items; factor loadings: 0.724–0.814), and “emotional adjustment for intellectual growth” (4 items; factor loadings: 0.688–0.899). Figure 2 shows the reduced version of the ECS-IN scale (18 items).

The researchers further used multiple fit indices to test the goodness of fit of the reduced version (18 items) of the ECS-IN scale, including the chi-square divided by the degrees of freedom (*χ*^2^/*df* ratio), Tucker–Lewis index (TLI), comparative fit index (CFI), and root mean square error of approximation (RMSEA). Being a test of statistical significance, to measure the goodness of fit, a *χ*^2^/*df* ratio value less than 3 with *p* values > 0.05 indicates little discrepancy between the observed values and expected values; a value less than 2 with *p* values > 0.05 indicates an acceptable discrepancy between the observed and expected values and hence an acceptable model fit [52,53]. As an incremental fit index, the Tucker–Lewis index (TLI) indicates the discrepancy between the chi-square value of the hypothesized model and the value of the null model. A bigger TLI value results in a better goodness of fit for the model [54]. However, in order to be considered an acceptable model fit, the TLI should be at least 0.90; a TLI of 0.95 is considered excellent [55]. As a corrected version of the relative noncentrality index, a CFI value larger than 0.90 is regarded as an acceptable model fit, and a value of 0.95 is an excellent model fit [56]. The RMSEA index refers to the discrepancy between the observed and the hypothesized covariance matrix per degree of freedom in the model [57]. An RMSEA index smaller than 0.08 is regarded as an acceptable model fit, and an RMSEA index smaller than 0.05 is an excellent model fit [56]. Table 3 shows the results of the goodness-of-fit indices of the reduced version (18 items) and the first version (29 items) of the ECS-IN scale—first version (29 items): *χ*^2^/*df* ratio = 3.277 (*p* = 0.000), TLI = 0.836, CFI = 0.849, RMSEA = 0.098; reduced version (18 items): *χ*^2^/*df* ratio = 1.090 (*p* = 0.246); TLI = 0.996; CFI = 0.997; RMSEA = 0.020. The three factors retained in the first version (29 items) of the ECS-IN accounted for 67.803% of the variance; the three factors retained in the reduced version (18 items) accounted for 70.274% of the variance. 

#### 3.2.2. Reliability

To examine the ECS-IN scale’s stability and internal consistency within each factor of the ECS-IN scale, the researchers used Cronbach’s alphas and composite reliabilities (CR), with values higher than 0.70 being considered acceptable values [42,43]. The Cronbach’s alphas for the three factors and for the overall scale of the first version (29 items) were 0.960 (“emotional identification as self-healing”), 0.938 (“emotional release for compensation”), 0.912 (“emotional adjustment for intellectual growth”), and 0.971. The Cronbach’s alphas for the three factors and for the overall scale of the reduced version (18 items) were 0.930, 0.907, 0.888, and 0.946, respectively; the composite reliability coefficients were 0.927, 0.908, 0.890, and 0.968, respectively. The Cronbach’s alphas and CR values were all higher than 0.70, the minimum acceptable value [58]; hence, both the first version (29 items) and reduced version (18 items) of the ECS-IN scale demonstrated fairly satisfactory reliabilities in assessing participants’ emotional catharsis through illness narratives (see Table 4).

#### 3.2.3. Convergent Validity

To further demonstrate the consistency of the reduced version (18 items) of the ECS-IN scale, the researchers tested the convergent validity of the scale, which was accessed by the AVE values and the composite reliabilities [42,43]. For adequate convergent validity, the AVE values should be not less than 0.50 and should be smaller than the composite reliabilities [42,43]. As in Table 5, among the AVE values of the three factors, the AVE value of “emotional identification as self-healing,” “emotional release for compensation,” and “emotional adjustment for intellectual growth” reveal acceptable convergent validities (0.645, 0.586, and 0.672, respectively), with the recommended cutoff point of 0.50 [45]. All the AVE values are lower than the composite reliabilities (0.927, 0.908, and 0.890, respectively; see Table 4 and Table 5.

#### 3.2.4. Discriminant Validity

Discriminant validity is confirmed when the √AVE is higher than the correlation coefficient (*r*) between factors [37]. The results of AVE, √AVE, and the correlation coefficients (*r*) between factors are shown in Table 5. The results show that the criterion is met with regard to the discrimination between “emotional identification as self-healing” and “emotional release for compensation” (√AVE = 0.803 and 0.766, respectively; *r* = 0.678), between “emotional release for compensation” and “emotional adjustment for intellectual growth” (√AVE = 0.766 and 0.820, respectively; *r* = 0.513), and between “emotional identification as self-healing” and “emotional adjustment for intellectual growth” (√AVE = 0.803 and 0.820, respectively; *r* = 0.651).

The above validity and reliability results demonstrate that the reduced version (18 items) as well as the first version (29 items) of the ECS-IN scale can be adopted as formal scales to measure the catharsis effect on medical professionals or students through illness narratives. The factors and items of the reduced version (18 items) of the ECS-IN scale are shown in the Appendix A.

## 4. Discussion

This study aimed to construct a comprehensive and psychometrically adequate scale measuring the emotional catharsis effect of illness narratives (ECS-IN) on medical professionals or students in order to provide them relief, or catharsis, surrounding matters of death and dying as well as the comfort that may come from finding meaning in human suffering. While testing the psychometrical properties of the ECS-IN scale, the researchers combined the techniques of EFA and CFA, performing the EFA on the sample of healthcare students to examine the latent variable structure of the dataset and then conducting the CFA on the sample of healthcare professionals to verify whether the factorial structure and factor-loading pattern fitted in the medical practice context.

The study first used the EFA to create a preliminary and longer version of the ECS-IN scale, which was composed of 29 items and three factors, explaining 67.803% of the variances. The study also used the promax rotation to obtain the greatest possible variance, usually lying in the first factor [40], “emotional identification as self-healing.” The three factors were “emotional identification as self-healing” (12 items), “emotional release for compensation” (10 items), and “emotional adjustment for intellectual growth” (7 items). The participants scored the highest on the “emotional adjustment for intellectual growth” subscale (mean = 6.426; 44.984 ÷ 7), followed by “emotional identification as self-healing” (mean = 5.719). The participants scored the lowest on the “emotional release for compensation” subscale (mean = 5.541). The average item scores on the three factors imply that the participants agree that illness narratives can help them emotionally adjust and hence reach intellectual growth. While emotionally identifying with those in illness narratives, they can achieve emotional release and further restore themselves. Additionally, while empathizing with the inner anxiety, fear, or negative feelings of those suffering, they can unleash their own negative feelings and thus find relief. The factor loadings on each single factor of the ECS-IN were ranged between 0.620 and 0.901, meeting the cutoff value of 0.50, with a value greater than 0.50 being considered adequate and a value greater than 0.70 being considered good [42]. Therefore, it can be concluded that each item in the first version (29 items) of the ECS-IN scale is considered appropriate for measuring the factor. The Cronbach’s alphas for the three subscales and the overall scale were ranged between 0.92 and 0.97.

While the researchers further used the CFA to examine the fitness of the first version (29 items) of the ECS-IN scale and the underlying latent factor structure of the ECS-IN scale, the study reduced the preliminary 29 items to 18 items, eliminating items 39, 42, 36, and 52, and 47 in factor 1, “emotional identification as self-healing”; items 11, 12, and 6 in factor 2, “emotional release for compensation”; and items 45, 20, and 22 in factor 3, “emotional adjustment for intellectual growth.” Thus, a shorter version of the ECS-IN scale was developed with only 18 items: “emotional identification as self-healing” (7 items), “emotional release for compensation” (7 items), and “emotional adjustment for intellectual growth” (4 items).

The factor loadings for each item were in the range between 0.688 and 0.899, indicating acceptable factor-loading values [42]. The researchers also used a variety of fit indices to investigate how well the reduced version of the scale (18 items) fit the data. As shown in Table 3, the indices of the first version (29 items) of the ECS-IN scale indicate almost acceptable model fits in *χ*^2^/*df* ratio (3.277; *p* value = 0.00), TLI (0.836), CFI (0.849), and RMSEA (0.098). However, in the reduced version (18 items) of the ECS-IN scale, the model fits all the fit indices, including *χ*^2^/*df* ratio, TFI, CFI, and RMSEA. Compared with the good-fit indices in the first version (29 items), the good-fit indices in the reduced version (18 items) of the ECS-IN scale demonstrates better goodness of fit, with an increase of 0.160 and 0.148 in TLI and CFI and a decrease of 0.078 in RMSEA and a decrease of 2.187 in *χ*^2^/*df* ratio value, also with an insignificant *p*-value (*p* > 0.05). Moreover, in the reduced version (18 items) of the ECS-IN scale, the total explained variance is increased to 70.274% compared with the first version’s 67.803% of variance.

In terms of convergent validity among the three factors, the AVE values of “emotional identification as self-healing,” “emotional release for compensation,” and “emotional adjustment for intellectual growth” are all above the cutoff value of 0.5 and smaller than the composite reliabilities. As reported by Fornell and Larcker [59], the convergent validity of the factors can be confirmed when the AVE value is not less than 0.5, while the composite reliability value should be above 0.6, and the AVE values should be smaller than the composite reliabilities [42,43].

As for the discriminant validities, according to Fornell-Larcker [59], the square root values of the AVE (√AVE) for each factor should be higher than the correlation coefficients (*r*) between factors. Hence, the results proved the discriminant validity between “emotional identification as self-healing” and “emotional release for compensation,” between “emotional release for compensation” and “emotional adjustment for intellectual growth,” and between “emotional identification as self-healing” and “emotional adjustment for intellectual growth.”

As for the reliabilities of the reduced version (18 items) and first version (29 items) of the ECS-IN scale, the researchers used Cronbach’s alpha and composite reliability statistics. As in Cunha, Almeida, and Stackfleth’s research [60] and Fornell and Larcker’s research [59], the minimally acceptable reliability value is 0.70, with the value not less than 0.80 for higher reliabilities [50,51]. In both the models, all the Cronbach’s alpha values and composite reliability values were higher than 0.80, with some even higher than 0.90. Hence, it can be concluded that, for both the first version (29 items) and reduced version (18 items) of the ECS-IN scale, the internal consistencies of the subscales and the overall scale are considered high reliabilities [42,43,59,60].

The above validity and reliability results support the three-factor structure for both the first version (29 items) and reduced version (18 items) of the ECS-IN scale. In view of these findings, it can be concluded that both the reduced version (18 items) and the first version (29 items) of the ECS-IN scale can be taken as formal scales to measure the emotional catharsis effect on medical professionals or students through illness narratives, with the first version (29 items) as the longer form for medical students and the reduced version (18 items) as the shorter form for medical professionals.

Though the research results demonstrated that both the first version (29 items) and reduced version (18 items) of the ECS-IN scale have good or even excellent reliabilities and validities, it cannot be denied that the study may have some limitations in that participants may not give honest answers. In addition, the developed ECS-IN scale is grounded on Taiwanese cultural contexts and on illness narratives so as to help healthcare professionals and students reach a catharsis to unburden themselves of pity, fear, and emotional stress in clinical settings. Thus, for those interested in using the scale, while translating the scale into their native languages, they should take their cultural backgrounds and participants’ backgrounds into consideration in order to let participants can fully comprehend the items.

## 5. Conclusions

The findings proved that both first version (29 items) and reduced version (18 items) of the ECS-IN scale can be a reliable and valid instrument in measuring participants’ emotional catharsis through illness narratives. Future studies may consider developing instructional strategies and curriculum regarding the use of illness narratives to achieve emotional catharsis, using both the first version and reduced version of the ECS-IN scale to assess them.

## Figures and Tables

**Table 1:** Rotated factor loading and eigenvalues of the first version (29 items) for the ECS-IN.

Item	Factor 1: Emotional Identification as Self-Healing	Factor 2: Emotional Release for Compensation	Factor 3: Emotional Adjustment for Intellectual Growth
Factor 1: α = 0.960			
39	0.901		
38	0.812		
37	0.803		
34	0.798		
40	0.795		
32	0.750		
31	0.745		
42	0.720		
36	0.717		
33	0.700		
52	0.695		
47	0.690		
Factor 2: α = 0.938			
3		0.858	
1		0.853	
9		0.748	
5		0.739	
10		0.728	
11		0.698	
12		0.677	
6		0.666	
13		0.663	
14		0.623	
Factor 3: α = 0.912			
48			0.869
45			0.856
50			0.820
22			0.724
20			0.724
51			0.658
49			0.620
Eigen value	16.095	2.165	1.403
% of variance	55.500	7.465	4.839

Overall α = 0.971; total variance explained is 67.803%.

**Table 2:** Average item scores (*Mean*) and standard deviations (*S.D.*) on the ECS-IN.

Factor and Item	*Mean*	*S.D.*
I. Emotional Identification as Self-Healing	68.630	17.293
39. By emotionally identifying with the characters in illness narratives, I can relieve my stress and other negative feelings without fear of criticism or blame from others.	5.61	1.695
38. While empathizing with the misfortune of patients, patient families, and healthcare professionals, I can reach emotional release and satisfaction, hence restoring myself to calmness and normalcy again.	5.75	1.684
37. By emotionally identifying with the characters in illness narratives, I can release my own grief and grievances and hence restore myself.	5.92	1.716
34. I can release my accumulated dissatisfaction, resentment, and anger by emotionally identifying with the characters in illness narratives.	5.49	1.747
40. By emotionally identifying with the characters in the illness narratives, I can reveal my human vulnerability as much as I can to achieve spiritual liberation without fear of criticism.	5.51	1.742
32. The plots about the death of a loved one can help me release the sadness that I do not want to face, thereby purifying my soul.	5.75	1.777
31. Via the illness narrative, I can project my unbearable emotions, feelings, or traits to others, hence releasing my mental burden.	5.76	1.672
42. Through illness narratives, I can unleash my inner negative emotions to regulate my personal emotions.	5.79	1.661
36. Through illness narratives, I can release my negative emotions and feelings to achieve self-healing and self-recovery.	6.10	1.725
33. Illness narrative can relieve my mental frustration.	5.21	1.833
52. Illness narratives can help me release negative emotions/feelings and heal wounds.	5.85	1.777
47. Through illness narratives, I can unleash my negative emotions and hence reconstruct my identity.	5.90	1.722
II. Emotional Release for Compensation	55.409	14.497
3. Illness narratives can help me release my inner anxiety and fear of death, separation, and loss of my loved ones due to disease.	5.72	1.813
1. Illness narratives can help me release my hidden negative emotions, such as fear, anxiety, and anger.	5.83	1.787
9. Illness narratives can help me speak out about my fear or other strong emotions I am embarrassed about and dare not express even in a safe place.	5.56	1.721
5. Illness narratives can help me reveal my inner fear and anxiety and somehow find a degree of pleasure in experiencing these emotions.	5.21	1.898
10. Through illness narratives, while empathizing with the misfortune of the characters, I also achieve emotional relief and release.	5.62	1.807
11. While feeling sad and frightened about the misfortune mentioned in the illness narratives (for example, unexpected death), I also obtain emotional release and satisfaction.	5.26	1.866
12. As for disorderly behavior mentioned in the illness narratives (for example, emergency department violence or hospital violence), somehow, I also find emotional release and satisfaction when I feel frightened and sad.	5.03	1.911
6. Through the illness narratives, I can unleash my emotions (for example, through a big cry) and find relief from sorrow and grief.	5.78	1.898
13. Illness narratives can help me securely express/discharge my negative emotions caused by trauma.	5.62	1.701
14.Through illness narratives, I can freely express my inner feelings and release my emotions.	5.77	1.702
III. Emotional Adjustment for Intellectual Growth	44.984	9.110
48. Through the illness narratives, I can come to terms with the transience of life and understand the meaning of life.	6.77	1.620
45. As I release my emotions through the illness narratives, I realize that life, aging, illness, and death are a part of the human experience, and I try to coexist with them.	6.58	1.607
50. Through the illness narratives, I can recognize the uncertainty and limitations of medical science and deal with them.	6.34	1.643
22. In the process of illness narratives, I can assess the behavior, attitude, and treatment that I may choose when facing the same predicaments or dilemmas as the characters in the narratives.	6.30	1.538
20. Through the illness narratives, I constantly reflect upon the dilemmas of patients, patients’ families, or healthcare professionals in order to achieve cognitive growth and understanding.	6.41	1.547
51. After releasing my emotions, the illness narratives further help me clarify the problems encountered by using a rational and positive attitude to deal with the immediate predicament.	6.32	1.646
49. Although the relationship between healthcare professionals and patients cannot be improved immediately, by illness narratives, I can face patients, patients’ families, and other healthcare professionals with a more appropriate emotional response.	6.26	1.646

**Table 3:** Goodness-of-fit indices for the first version (29 items) and the reduced version (18 items) of the ECS-IN scale.

	$\chi^{2}$	*df*	χ2df	*p*	TLI	CFI	RMSEA	% of Variance
First Version of the Scale (29 items)	1225.637	374	3.277	0.000	0.836	0.849	0.098	67.803%
Reduced Version of the Scale (18 items)	116.654	107	1.090	0.246	0.996	0.997	0.020	70.274%

Note: *df*, degree of freedom; TLI, Tucker–Lewis index; CFI, comparative fit index; RMSEA, root mean square error of approximation.

**Table 4:** Cronbach’s alphas and composite reliabilities of the first version (29 items) and the reduced version (18 items) of the ECS-IN.

	Reliability	Cronbach’s Alpha ECS-IN—First Version and Long Version of the Scale (29 Items)	Cronbach’s Alpha ECS-IN—Reduced Version and Short Version of the Scale (18 Items)	Composite Reliability ECS-IN—Reduced Version and Short Version of the Scale (18 Items)
Factor				
Emotional Identification as Self-Healing		0.960	0.930	0.927
Emotional Release for Compensation		0.938	0.907	0.908
Emotional Adjustment for Intellectual Growth		0.912	0.888	0.890
Overall		0.971	0.946	0.968

**Table 5:** AVE, √AVE, and correlation coefficients (*r*) between factors.

Factor	AVE	1	2	3
1. Emotional Identification as Self-Healing	0.645	**0.803**		
2. Emotional Release for Compensation	0.586	0.678 **	**0.766**	
3. Emotional Adjustment for Intellectual Growth	0.672	0.651 **	0.513 **	**0.820**

The values shown in bold are the square root of AVE (√AVE). ** *p* < 0.001.

## Abbreviations

EFA, Exploratory Factor Analysis; CFA, Confirmatory Factor Analysis; TLI, Tucker—Lewis Index; CFI, Comparative Fit Index; RMSEA, Root Mean Square of Approximation.

## Appendix A. The Factors and Items of Reduced Version (18 Items) of the ECS-IN Scale

Factor 1: Emotional Identification as Self-Healing

1. While empathizing with the misfortune of patients, patient families, and healthcare professionals, I can reach emotional release and satisfaction, hence restoring myself to calmness and normalcy again.
2. By emotionally identifying with the characters in illness narratives, I can release my own grief and grievances and hence restore myself.
3. I can release my accumulated dissatisfaction, resentment, and anger by emotionally identifying with the characters in illness narratives.
4. By emotionally identifying with the characters in the illness narratives, I can reveal my human vulnerability as much as I can to achieve spiritual liberation without fear of criticism.
5. The plots about the death of a loved one can help me release the sadness that I do not want to face, thereby purifying my soul.
6. Via the illness narrative, I can project my unbearable emotions, feelings, or traits to others, hence releasing my mental burden.
7. Illness narrative can relieve my mental frustration.

Factor 2: Emotional Release for Compensation

8. Illness narratives can help me release my inner anxiety and fear of death, separation, and loss of my loved ones due to disease.
9. Illness narratives can help me release my hidden negative emotions, such as fear, anxiety, and anger.
10. Illness narratives can help me speak out about my fear or other strong emotions I am embarrassed about and dare not express even in a safe place.
11. Illness narratives can help me reveal my inner fear and anxiety and somehow find a degree of pleasure in experiencing these emotions.
12. Through illness narratives, while empathizing with the misfortune of the characters, I also achieve emotional relief and release.
13. Illness narratives can help me securely express/discharge my negative emotions caused by trauma.
14. Through illness narratives, I can freely express my inner feelings and release my emotions.

Factor 3: Emotional Adjustment for Intellectual Growth

15. Through the illness narratives, I can come to terms with the transience of life and understand the meaning of life.
16. Through the illness narratives, I can recognize the uncertainty and limitations of medical science and deal with them.
17. After releasing my emotions, the illness narratives further helped me clarify the problems encountered by using a rational and positive attitude to deal with the immediate predicament.
18. Although the relationship between healthcare professionals and patients cannot be improved immediately, by illness narratives, I can face patients, patients’ families, and other healthcare professionals with a more appropriate emotional response.
